# Computational Framework Explains How Animals Select Actions with Rewarding Outcomes

**DOI:** 10.1371/journal.pbio.1002035

**Published:** 2015-01-06

**Authors:** Janelle Weaver

**Affiliations:** Freelance Science Writer, Carbondale, Colorado, United States of America

## Abstract

A new model of how the brain learns beneficial behavior from rewarding outcomes emphasizes the importance of the striatum, replicates experimental data, and raises new questions about neurological disorders. Read the Research Article.

A key component of survival is learning to associate rewarding outcomes with specific actions, such as searching for food or avoiding predators. Actions are represented in the cortex—the brain's outer layer of neural tissue—and rewarding outcomes activate neurons that release a brain chemical called dopamine. These neuronal signals are sent to the striatum—the input station for a collection of brain structures called the basal ganglia, which play an important role in action selection. Collectively, this evidence suggests that dopamine signals change the strength of connections between cortical and striatal neurons, thereby determining which action is appropriate for a specific set of environmental circumstances. But until now, no model had integrated these strands of evidence to test this widely held hypothesis.

In a study published this week in *PLOS Biology*, University of Sheffield researchers Kevin Gurney and Peter Redgrave teamed up with Mark Humphries of the University of Manchester to build a computational model that shows how the brain's internal signal for outcome changes the strength of neuronal connections, leading to the selection of rewarded actions and the suppression of unrewarded actions. By bridging the gap between the intricate subtleties of individual neuronal connections and the behavior of the whole animal, the model reveals how several brain signals work together to shape the input from the cortex to the basal ganglia at the interface between actions and their outcomes.

The researchers developed a network model of the whole basal ganglia based on previous electrophysiological studies that investigated the activity of two types of dopamine-responsive cells called D1 and D2 striatal medium spiny neurons. In addition to this action selection model, they developed an independent plasticity model by incorporating experimental data from a previous study to show how the strength of neuronal connections, called synapses, is affected by three factors: the timing of neuronal activity, the type of medium spiny neuron, and dopamine level. Then they linked the two models, testing whether plasticity rules at single synapses between cortical and striatal neurons could give rise to the predicted changes in the activity of the two types of medium spiny neurons, resulting in successful learning of the association between actions and outcomes.

Achieving a remarkable convergence between vastly different scales of space and time, the computational framework not only replicates experimental data on cortico-striatal plasticity but also accounts for behavioral data on learning the association between actions and outcomes (Fig. 1). The model revealed that the relative strength of cortical inputs that represent different possible actions to the two populations of medium spiny neurons determines whether an action is selected or suppressed. Moreover, the timing of neuronal activity, the type of medium spiny neuron, and dopamine level are necessary for generating neuronal activity patterns that result in successful learning.

**Figure 1:**
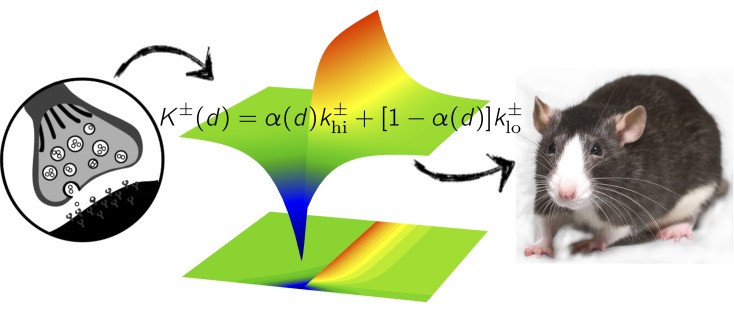
Learning how to make decisions: a computational model bridges the gap between the intricate subtleties of individual neuronal connections and the behavior of the whole animal. *Image credit: Left: Iwan Gabovitch, openclipart; Center: Kevin Gurney; Right: Alexey Krasavin, Flickr.*

According to the dominant conceptual model of the basal ganglia, D1 and D2 medium spiny neuron populations project through two competing pathways that either promote or suppress an action. This model has been used to explain the motor symptoms of Parkinson's disease, Huntington's disease, and other neurological disorders associated with basal ganglia dysfunction. However, the new network model revealed that D1 and D2 medium spiny neurons coding the same action cooperate to produce optimal action selection, suggesting that the dominant model is not true and needs to be reevaluated.

Taken together, the results of the study provide strong support for the hypothesis that cortical inputs to dopamine-releasing neurons in the striatum are crucial for learning the association between action and outcome. Moving forward, the model provides a common framework in which to place new findings on all aspects of learning from outcomes. In the clinical realm, it could also reveal novel insights into the mechanisms behind motor disorders and shed light on abnormal learning related to diseases such as addiction. Moreover, the work highlights the importance of the striatum, not just the cortex, in high-level learning that is crucial for survival, possibly explaining why the basal ganglia are highly evolutionarily conserved across vertebrate species.

**Gurney KN, Humphries MD, Redgrave P (2015) A New Framework for Cortico-Striatal Plasticity: Behavioural Theory Meets In Vitro Data at the Reinforcement–Action Interface.** doi:10.1371/journal.pbio.1002034

